# Barriers to medication adherence in a rural-urban dual economy: a multi-stakeholder qualitative study

**DOI:** 10.1186/s12913-021-06789-3

**Published:** 2021-08-12

**Authors:** Jacqueline Xu, Mengxi Zhao, Athina Vrosgou, Natalie Chin Wen Yu, Chelsea Liu, Han Zhang, Chunxi Ding, Noelle Wyman Roth, Yuesong Pan, Liping Liu, Yilong Wang, Yongjun Wang, Janet Prvu Bettger

**Affiliations:** 1 Trinity College of Arts and Sciences, Duke University, Durham, NC USA; 2 Department of Neurology, Beijing Tiantan Hospital, Capital Medical University, Beijing, China; 3 Institute of Social and Cultural Anthropology, University of Oxford, Oxford, UK; 4 Stanford Prevention Research Center, Stanford University School of Medicine, Palo Alto, CA USA; 5 Harvard T.H. Chan School of Public Health, Harvard University, Boston, MA USA; 6 Social Science Research Institute, Duke University, Durham, NC USA; 7 China National Clinical Research Center for Neurological Diseases (NCRC-ND), Beijing, China; 8 Department of Orthopaedics, Duke University School of Medicine, Durham, NC USA; 9 Duke Global Health Institute, Duke University, Durham, NC USA; 10 Duke-Margolis Center for Health Policy, Duke University, Durham, NC USA

**Keywords:** Developing Countries, Medication Adherence, Noncommunicable Diseases, Qualitative Research, Stroke

## Abstract

**Background:**

One of the most cost-effective treatments for secondary prevention of stroke and other non-communicable diseases is a long-term medication regimen. However, the complexities of medication adherence extend far beyond individual behavior change, particularly in low- and middle-income countries. The purpose of this study was to examine stakeholder perspectives on barriers to medication adherence for stroke patients in Beijing, China, identifying opportunities to improve care and policy in resource-constrained settings.

**Methods:**

We conducted a qualitative, phenomenological analysis of data obtained from 36 individuals. Participants were patients; caregivers; healthcare providers; and representatives from industry and government, purposively selected to synthesize multiple perspectives on medication management and adherence for stroke secondary prevention in Beijing, China. Data was analyzed by thematic analysis across iterative coding cycles.

**Results:**

Four major themes characterized barriers on medication adherence, across stakeholders and geographies: limitations driven by individual patient knowledge / attitudes; lack of patient-provider interaction time; lack of coordination across the stratified health system; and lack of affordability driven by high overall costs and limited insurance policies.

**Conclusions:**

These barriers to medication management and adherence suggest opportunities for policy reform and local practice changes, particularly for multi-tiered health systems. Findings from this study in Beijing, China could be explored for applicability in other low- and middle-income countries with urban centers serving large geographic regions.

**Supplementary Information:**

The online version contains supplementary material available at 10.1186/s12913-021-06789-3.

## Background

Non-communicable diseases (NCDs)—including cardiovascular diseases, hypertension, and diabetes—are the leading cause of deaths worldwide, responsible for 40.5 million lives each year. Low- and middle-income countries (LMICs) account for more than three-quarters of global NCD mortality [1]. Global efforts have called attention to the need to address NCDs and long-term adherence to appropriate medications, such as anti-hypertensives, insulin, and other common medicines [2]. However, supporting long-term medication adherence is multifaceted. National policy and local health systems have a critical role in improving access and other strategies that support health care professionals and their patients with adherence to essential medicines to effectively address NCD disease burdens [3].

The seemingly simple action of taking pills is, in fact, deceptively complex [4]. Patients must understand the need for the medications prescribed, interact with their provider to obtain initial and follow-up prescriptions, have a plan to continuously pick up or receive the medications, be able to pay for them, remember to take them, and feel motivated to consume their medication at the right time and in the right amounts. For some patients, this process could necessitate involvement of additional family members to obtain the medications or provide reminders. There is a significant amount of knowledge, coordination, and financial capital required for adherence to secondary preventative medications. Health systems working in a fragmented healthcare landscape struggle to provide such preventative care [5]. For LMICs, human and financial resource constraints compound these existing challenges [6, 7].

China is an upper-middle income country where NCDs, particularly cardiovascular diseases and associated risk factors, represent a significant burden. Stroke is the leading cause of death in China, followed by ischemic heart disease [8]. Medication regimens prescribed after stroke help patients prevent event recurrence [9]. However, many studies from China suggest low adherence of approximately 35 % for anti-hypertensives, 35 % for statins, and 27–31 % for diabetes mellitus related medication regimens [10–14]. The specific barriers to adherence are unclear, particularly with regard to the role of social services and health insurance in China. Like many LMICs, China struggles with an uneven distribution of healthcare resources across urban and rural areas [15, 16]. Within densely populated megacities like Beijing’s city centers, there are tertiary level hospitals dedicated to specialty and resource-intensive care. However, Beijing’s city outskirts lack the same level of resources, resulting in significant health disparities [17, 18]. It is important to understand the complexities of medication adherence in the context of this fragmentation to see how different factors can ultimately affect patient behavior and subsequent outcomes.

Systems of care have multiple levels. When applied to complex health problems, these levels include the patient / family, healthcare providers, health care service delivery systems, and national health policy [19]. This study explored the barriers to secondary prevention medication adherence after stroke, through the perspectives of stakeholders from patient to system level. We studied China’s Beijing for its potential generalizability to major Chinese cities and other regions with uneven resource distribution in South Asia, Africa, and South America. We hope the perspectives of patients, caregivers, healthcare providers, administrators, and industry can serve as a catalyst for focused discussions in similar regions and facilitate the creation of more effective policy and organizational level strategies.

## Methods

### Study design

We conducted a qualitative, phenomenological study to describe the barriers of medication adherence from the perspectives of individuals involved in different aspects of secondary prevention after stroke [20]. This research was performed in accordance with the Declaration of Helsinki. Ethics approval was obtained from the Human Research Ethics Committee of Tiantan Hospital in China and Institutional Review Board at Duke University in the United States (USA). All interviewees provided verbal informed consent and were informed their participation was voluntary.

### Participants

Adhering to secondary preventative medications requires involvement from multiple actors along a process, beginning with identifying a need for treatment, follow-up visits, and the act of taking the medication itself. Therefore, we sought to interview patients, family caregivers, healthcare providers, hospital administrators, representatives from the pharmaceutical industry, and a government official representing the region. The type of healthcare organization was important as China has a tiered system for healthcare with tertiary, secondary, and community hospitals that are located in the city center and city outskirts [21]. We identified and included hospital administrators in decision-making roles regarding patients’ medication prescriptions at hospital discharge. Inclusion criteria for healthcare provider participants were physicians and nurses directly involved in the care of stroke patients in a hospital or outpatient clinic setting. Participating providers subsequently helped identify potential patients and caregivers for this study. We included both stroke and transient ischemic attack (TIA) patients. When patients were deemed unfit to interview directly, such as those who had poor cognitive function or were critically ill, their caregivers served as the study participant on the patients’ behalf. Caregivers were required to have been the patient's primary caregiver and directly involved in all phases of care, from acute to follow-up settings. We sought balance in age among the patient / caregiver participants invited to participate, and half represented older adult patient experiences (age 65 and over); however, we did not select individuals based on the number of medications prescribed or ability to pay. We selected pharmaceutical company representatives who interacted with healthcare providers specifically for stroke secondary prevention medications. Overall, individuals were purposively selected based on principles of maximum variability in order to capture perspectives across hospital tiers and geographic regions [22]. Participants were recruited across all areas of Beijing (from city center to outskirts) until thematic saturation was reached. Fourteen individuals who were invited did not respond or declined to participate. The final study sample included interviews with 36 individuals.

### Procedures

Interviews followed a semi-structured guide exploring barriers and facilitators to secondary preventative medication management after stroke (see Additional File 1 for details.) This guide was the same for all stakeholder interviews, with minor wording changes for personalization when used with patients and caregiver participants. The guide was developed by an interdisciplinary team consisting of neurologists and health services researchers from China and the USA. The guide was forward- and back-translated from English to Mandarin and piloted prior to use.

All interviews were then conducted face-to-face in a location designated by participant preference, from June to October 2017. The semi-structured interviews were conducted by three trained bilingual researchers (JX, CL, ML), unaffiliated with any interviewees. Interviews were conducted in Mandarin, with the exception of one interview because of the participant’s English fluency. Twenty-five interviews were conducted with 36 individuals, each approximately one hour in duration. While 21 of the 25 interviews were conducted one-on-one, there were four interviews conducted with more than one participant. All interviews were audio-recorded, transcribed, and translated into English for analysis, with field notes made during each interview. Bilingual team members validated the translated scripts to ensure high data integrity. Any disagreements regarding translations were resolved by consensus.

### Data analysis

We used the Framework method for data analysis, a five-step qualitative technique for identifying prevailing themes among interviews [23, 24]. This approach supports management of large amounts of data when making comparisons within and between described experiences. In order to develop an analytic framework, team members (JX, CL, CD, HZ, NY, AV) began with line-by-line inductive coding on an initial subset of 13 interviews across stakeholder types. The same team members then coded the remaining 12 interviews to this framework, ensuring full capture of prominent themes. Each interview was coded by two different team members in NVivo v12.2 (QSR International, Victoria, Australia), and discrepancies were discussed among the research team with resolution by consensus. The framework that emerged showed four primary themes. These themes were further analyzed using ecological systems theory to identify levels of influence on patients' medication adherence [19]. This included consideration of themes at the patient, provider, healthcare system, and societal levels. Application of this theory allowed us to consider influence from the immediate environment (microsystem), connections between people (mesosystem), indirect environment (health system), and social, political and cultural norms (macrosystem). As a cross-sectional study of perspectives, we did not explore the chronosystem or changes over time and instead analyzed data to identify differences by geography.

## Results

Participant characteristics are summarized in Table 1. Participants represented a diverse range of roles and practice settings across the Beijing region.

**Table 1:** Characteristics of interview participants, total *n* = 36

Characteristic	n
**Role**	
Healthcare Administrator	14
Healthcare Provider	10
Patient or Caregiver	8
Private Sector Employee	3
Government Official	1
**Hospital Tier**	
Community (Tier 1A, 1B, 1C)	20
Secondary (Tier 2A, 2B, 2C)	7
Tertiary (Tier 3A, 3B, 3C)	5
N/A (non-healthcare center)	4
**Geographical Region**	
City Center	16
City Outskirts	16
N/A (jurisdiction over entire municipality)	4

Despite the variability in role and region, participant interview data revealed consistency of perspectives on barriers to medication adherence that cut across four levels: individual patient, patient-provider interactions, health system, and societal. Table 2 summarizes the emerging themes from coded interviews.

**Table 2:** Emerging themes on barriers to medication adherence among stakeholders in Beijing, China (total *n* = 36)

Level	Theme / Subtheme	n
**Individual patient**	*Lack of medical knowledge* - Misperceptions on preventative medications - Misguided fears on risk of side effects	33
	*Attitudes of apathy* - Symptoms of depression and anxiety - General resignation / “giving up” on health-seeking behavior	27
**Patient-provider interaction**	*Shortage of providers* - Limited time for individualized patient education - Heavy patient caseloads / lack of doctor-patient relationship	27
**Health systems**	*Lack of coordinated follow-up* - Lack of centralized health data management system - Limited communication across tiered providers	33
	*Resourcing and geographic spread between tiered providers*	33
**Societal**	*Ability to pay / affordability* - Cost of long-term medication usage - Additional cost of associated services	29
	*Insurance policies*	32

At each level of socioecological influence, emerging theme(s) characterized the perceived barriers on the ability of patients to adhere to their secondary medication prescription regimens after a stroke, summarized as follows: (1) limitations driven by individual patient knowledge / attitudes; (2) lack of patient-provider interaction time; (3) lack of coordination across the stratified health system; and (4) restrictions on affordability driven by high overall costs and insurance policies. While systems theory would suggest that factors at macro-levels of society such as organization of care and affordability would have a weaker influence on adherence, our participants felt strongly that current systems were directly contributing to poor adherence and have greater population impact. More barriers were perceived for patients from rural areas, and challenges introduced due to the poor bridging of care and medication access between urban to rural settings were intensified for factors at the health system and societal levels.

### Level 1. Individual patient-related barriers

#### Theme 1.1 Lack of medical knowledge

Patients’ gaps in knowledge and lack of medical experience were perceived as factors that provoked an unwillingness to take medication until prompted by symptoms. Without the knowledge nor experience, patients were not well-equipped to change their behaviors.

*I had an episode [of stroke]. At the time I didn’t really care about it…I had never taken medication over my many years of work. I had never been hospitalized.*

Patient, Community Hospital

*Actually I think it’s that their [patient’s] understanding is insufficient, their understanding of the long-term treatment goals is insufficient…They only see this time, “I’m good I don’t have any symptoms. I’m good, so I will not take medicine or do follow-up examination.”*

Administrator, Community Hospital

Gaps in knowledge were also described as leading to increased fear of side effects, commonly reported as barriers to adherence. Some patients held on to misguided information and beliefs.

*For example, if a patient has had bleeding before, then if you encourage him to take aspirin again, he will feel that this will definitely cause him to bleed.*

Administrator, Tertiary Hospital

#### Theme 1.2 Attitudes of apathy

In addition to gaps in patient medical knowledge, interview participants also commonly referred to the onset of psychological disorders, such as depression and anxiety, as an additional barrier to adherence behavior. Stroke can be a debilitating disease that greatly impacts both patients’ physical and mental health, decreasing outcomes along quality-of-life metrics.

*After suffering a stroke, besides the impact of the disease on their physical bodies, they may have psychological symptoms of depression or anxiety. They may also develop a sense of resignation or self-loathing towards their lives. They may not want to be treated.*

Administrator, Community Hospital

Outside of formal diagnosis, providers and administrators commonly noted how feelings of resignation and “giving up” influenced patients to become unmotivated or disinterested in rehabilitation. Ultimately, all data on patient experience suggested that health knowledge, attitudes, and experience influence medication adherence behaviors.

*But the reason why adherence level is still low in villages, it’s because their [health] consciousness is lacking, [patients] feel that there are times when it doesn’t matter whether they take their medication or not, so they choose not to.*

Clinical Provider, Community Hospital

### Level 2. Patient-provider interaction-related barriers

#### Theme 2.1 Shortage of providers

Some participants reported that gaps in patient knowledge were a result of missed opportunities on the physicians’ part, particularly in the transmission of information and coaching from medical provider to patient. Providers and administrators often reported too little time to provide education, resulting in patients leaving appointments without a clear understanding of their medications. Ultimately, these factors led to patients lacking knowledge and understanding that would otherwise allow them to adhere to their treatment plans. As a result, many interviewees reported patients self-modifying their treatment in a misguided manner.

*Because there are fewer doctors but more patients in China, we do not have enough time to explain their conditions to them….I hope we can also have services like family doctors, but the family doctor system is hard to fulfill, because of the limited resources of health providers, and they do not have time and energy to do this.*

Administrator, Community Hospital

Participants often described these time constraints in the context of heavy patient caseloads. Overloaded providers not only lacked time for patient education, but were also unable to develop doctor-patient relationships and encourage adherence behaviors through coaching. However, when participants did describe the successful formation of a doctor-patient relationship, it was nearly always in the context of a community hospital setting.

*When you are hospitalized, you have to go ask about the test results, [the providers] don’t write [the results] down. I go ask about what different things mean…Doctors are different. Sometimes they will explain…. After [test results] were done, usually, I go find [name of doctor in community hospital], usually I go to her for any checkups on my tests.*

Patient, Community Hospital

### Level 3. Health systems-related barriers

#### Theme 3.1 Lack of coordinated follow-up

Patients and providers alike expressed how communication and health data are not well-managed across providers. The burden of bringing medical histories to the next clinical encounter falls on the patient, yet patients often lack the health literacy needed to appropriately describe complexities of their condition between providers.

*There isn’t any electronic network for the hospital system. The patient, if, for example, I give a prescription, and the other doctor wants to see it, the patient has to hand the paper prescription to the doctor*.

Clinical Provider, Tertiary Hospital

*So this check-up [at the hospital] I discovered that it’s one-off. The doctors keep changing and there is no continuity. I don’t really understand the situation, but just now, we came for follow-ups. The doctor that came…he said something and [I could see] he didn’t understand what was going on.*

Caregiver, Community Hospital

Coordination is also lacking between care provided at tertiary, secondary, and community hospitals, exacerbating issues with patient medication adherence. This system of healthcare stratification requires patients to visit community hospitals for non-emergency care, even though these community-level hospitals may lack the medications and diagnostics they need.

*In reality, there is the need for more linking between hospitals. The recent health care reform required better connectedness between secondary/tertiary and community hospitals, but this wasn’t accomplished entirely. So now, for example, in this community, there isn’t too much contact between patients in the community hospitals and this hospital. We just each do our own thing*.

Clinical Provider, Secondary Hospital

#### Theme 3.2 Resourcing and geographic spread between tiered providers

Barriers to coordination and quality differences between healthcare strata (community, secondary, and tertiary hospitals) place additional burden on rural patients, particularly those with chronic disease management needs. Rural-dwelling patients must contend with longer distances to the secondary and tertiary hospitals located in the city center or select to be managed by local clinics. This inconvenience of distance and time sets up additional barriers for rural patients who need care from more specialized hospitals.

*It’ll definitely be like this because the large hospitals are very hard [to travel and navigate to]…they’re good but they’re so expensive, so [patients] won’t often go, and every time they go they can only get up to one to two weeks’ worth of medication.*

Pharmaceutical Representative

*[Follow-up] is the difficult part of the process. If it were me, I wouldn’t go to the main hospital for a re-examination. It’s really very taxing.*

Provider, Community Hospital

### Level 4. Societal-related barriers

#### Theme 4.1 Ability to pay / affordability

Affordability of medication and health services was the second-most commonly reported barrier to patient medication adherence. Since secondary preventative medications are often prescribed for long-term use as a mix of different types of drugs, many participants described challenges committing to the high costs of their medication regimens.

*And then there are people without health insurance, and they have even worse compliance…[However,] aspirin is affordable, it’s cheap, and so they will use it. But metformin, statins, medication that costs more, they won’t use it.*

Administrator, Community Hospital

Further, participants also detailed “hidden costs” of adherence, given multiple follow-up visits were often required to adjust and check the status of their prescribed regimen. These costs further exacerbate the existing financial barriers for patients to adhere to their secondary prevention medication regimen.

*I also felt angry. Originally, you wouldn’t pay [too much] more to see the doctor, but this expenditure is not insignificant, and when I was stressed, I went to try different medical treatments. I have a lot I haven’t been able to claim; I’ve spent all my savings.*

Patient, Community Hospital

#### Theme 4.2 Insurance policies

Barriers are compounded for patients living in rural areas not only because of geographic access, but also differences in insurance policy and coverage. Participants often described insurance, specifically coverage restrictions, as intimately tied to affordability challenges.

*3000 RMB [cap of New Rural Cooperative Scheme health insurance reimbursements, ~$443 USD] per year, if they have other conditions like high blood pressure, diabetes or other chronic diseases, the percentage [spent on] medication will be very high, so it’s possible that they don’t consume stroke medication.*

Provider, Community Hospital

*Another aspect is that even if this group of [rural] patients has insurance…the insurance places a lot of restrictions on the patient. For example, this type of commonly used medication, you can only take 2 weeks’ supply…[the patient] needs to keep coming for medical consultations [to refill their medication]*.

Administrator, Tertiary Hospital

These issues of affordability and gaps in patient knowledge interact, since rural patients are doubly disadvantaged by lower education levels and lower income. Both are key barriers, although there remain differing opinions as to the relative contributions of each to the larger problem of low medication adherence.

## Discussion

Stroke burden in China is substantial, with more than 11 million stroke survivors alive at any given time [25]. Secondary prevention is the most efficacious strategy for the prevention of stroke recurrence, and our study identified addressable barriers. In a complex and dynamic megacity like Beijing with a widening rural-urban divide, we found barriers to medication adherence after a stroke across four socioecological levels and summarized as follows: (1) limitations driven by individual patient knowledge / attitudes; (2) lack of patient-provider interaction time; (3) lack of coordination across the stratified health system; and (4) restrictions on affordability driven by high overall costs and insurance policies. Findings suggest a multi-faceted strategy is needed to improve medication adherence, including improving patient education during follow-up care, increasing opportunities for patient-provider interaction, and aligning national insurance plans to incentivize care coordination. As many other counties have urban centers servicing surrounding rural regions for acute health care needs, our findings raise awareness for the need to support patients being managed across multiple hospital or clinic tiers. The gaps may be more pronounced for patients transitioning from an acute tertiary hospital to home, where continued self-management is expected at the community-level with constrained healthcare resources.

Study participants reported patient-level medical experience as the most influential factor for medication adherence. Patients lacking prior experience managing stroke or other chronic diseases did not fully understand the importance of secondary prevention, in which medication regimens need to be followed even if patients are asymptomatic. Studies among stroke patients, caregivers, and primary care providers in high-income countries like France and the United Kingdom support our findings [26, 27]. A meta-analysis of patient beliefs about medication across multiple illness conditions found similar results regarding the importance of patient-level knowledge and experiences [28]. However, our study also identified an opportunity to shape patients’ beliefs by improving the quality and quantity of provider interactions. Studies among high and low adherers to medication regimens for stroke prevention and other chronic conditions have similarly identified provider resources and time as critical elements to adherence [25, 29].

Both patient and provider participants expressed the inability to fully discuss treatment adherence and answer patient questions during medical visits. Recognizing that acute hospital lengths of stay for stroke are longer in LMICs than in high-income countries, enhanced patient-provider interaction specific to medications could begin prior to hospital discharge [30, 31]. This approach allows information to be repeated in interactions on multiple days. Discussions held on medication prioritization and financial capacity before discharge can prevent prescriptions that families do not plan to fill [32]. Patient and caregiver understanding can be assessed, addressed, and reassessed. Interactions with pharmacies at hospital discharge could be opportunities for enforcing prior messages. Subsequently, follow-up outpatient clinic visits would no longer be the first opportunity to discuss secondary prevention and the importance of medication adherence. This may also reduce the barriers associated with care coordination specific to stroke prevention when transitioning from acute to primary care and urban to rural healthcare services. A study in Huangzhong, China illustrated that although primary care practitioner models are becoming more common in China, they have not led to larger structural improvements in care coordination [33]. Organizational changes that span the care continuum are required to support a more patient-centered approach that fosters engagement among clinicians from across the system. Future investments in China and LMICs need to address health system building blocks, creating capabilities for electronic health data and workforce capacity in particular [34].

Cost was the second most common barrier to medication adherence after stroke. In 2016, out-of-pocket payments comprised 28.8 % of total health expenditure in China. This is a dramatic decline in comparison to 60 % out-of-pocket spending in 2001 and was driven by expanded government health insurance coverage and investments. However, 44.1 % of impoverished households in China still cited illness as the driver of their spending in 2015 [35]. Low coverage rates of rural insurance schemes coupled with high-cost health services after inpatient stroke care can leave patients drained and wary of further health spending after acute events. As such, even lower-cost interventions like secondary preventative medications remain unaffordable. In China, clopidogrel treatment rates in patients with the lowest versus highest socioeconomic status were 6.7 and 34.2 %, respectively; treatment rates for statins follow a similar pattern of 41.7 and 75 % [36]. China and other LMICs need to continue providing wider insurance coverage or establish a national essential medication system to reduce the catastrophic costs of stroke.

Several limitations of this study should be recognized. While qualitative design enabled identification of complex barriers across individual to system levels, qualitative research remains subject to bias from participant selection and investigator involvement [37]. To enhance the validity and reliability of our work, we employed several techniques including word-for-word transcription and back-validation of transcripts to ensure accurate capture of interviewee responses, and involvement of both medically and non-medically trained interviewers. In addition, social desirability bias may have motivated respondents to answer to please the interviewer. We attempted to mitigate this bias by ensuring that interviewers were not affiliated with participants and that no prior relationship was established.

## Conclusions

Secondary prevention medications are widely acknowledged as one of the most cost-effective methods to improving outcomes for stroke and other NCD patients. This study identified four major barriers to stroke secondary prevention medication adherence: patient knowledge and attitudes, patient-provider interaction time, healthcare fragmentation, and policies influencing affordability. As this research illustrates, organizational and system factors can and often do hinder adoption of this seemingly simple patient behavior. Our study highlights the importance of developing multi-level strategies to support patient medication management for stroke and other NCDs.

Our research is descriptive and hypothesis-generating. We hope this study can help inform future research in LMICs with similar health system structures that span from acute-to-community care across urban and rural geographies and stimulate discussions to generate policies that influence healthcare coordination, provider time, and patients’ access and affordability. There is an opportunity for greater partnership and collaboration between the public and private sectors, as well as between healthcare professionals and patients to address barriers to medication adherence.

## Supplementary Information

**Additional file 1.** Interview Guide.

## Data Availability

The datasets generated and analyzed during the current study are not publicly available—given a need to protect individual participant data—but are available from the corresponding author on reasonable request.
