# Supplementary material for: Barriers to medication adherence in a rural-urban dual economy: a multi-stakeholder qualitative study
Source: BMC Health Serv Res. 2021 Aug 12;21:799. doi: 10.1186/s12913-021-06789-3 (PMC8359298; doi:10.1186/s12913-021-06789-3)
Supplement: Supplementary file 1 — Additional file 1. Interview Guide. [file 12913_2021_6789_MOESM1_ESM.docx]

**Additional File 1**

Barriers to medication adherence in a rural-urban dual economy: A multi-stakeholder qualitative study (Xu et al., 2021).

Interview Guide (English version)

1. Describe your experience working with stroke or TIA patients.

(*For patients and caregivers:* Describe your experience with stroke or TIA.)

1. What do you think are the largest unmet needs in post-acute care for stroke patients in Beijing (with regards to secondary prevention) (e.g., health education to encourage lifestyle changes, medication adherence support)?
2. What is your perspective on existing levels of stroke patient medication adherence?

(*For patients and caregivers:* How would you evaluate your current level of medication adherence?)

1. What are the barriers and facilitators for stroke patients to obtain / fulfill their secondary prevention prescriptions (e.g., time for follow-up visits, distance from healthcare facility, insurance / cost, types of medications in their regimen?)
   1. Do you think stroke patients and / or caregivers have the ability to understand and follow their secondary prevention medication regimen?

(*For patients and caregivers:* Did you feel confident and comfortable following your secondary prevention medication regimen?)

1. What are the barriers and facilitators to stroke patients then taking their secondary prevention medications (e.g., side effects, lacking refill supply, belief there was no longer a need to take medicines)?
2. How do, if at all, these barriers and facilitators vary for different types of stroke patients in Beijing (e.g., place of residence, age, disease severity)?
3. What is your overall level of satisfaction with the current state of post-acute care for stroke patients in Beijing?
